# Supplementary material for: Micronutrient Status of Recreational Runners with Vegetarian or Non-Vegetarian Dietary Patterns
Source: Nutrients. 2019 May 22;11(5):1146. doi: 10.3390/nu11051146 (PMC6566694; doi:10.3390/nu11051146)
Supplement: Supplementary file 1 [file nutrients-11-01146-s001.pdf]

nutrients

Article – Supplementary Material

Micronutrient status of recreational runners with vegetarian or non-vegetarian dietary patterns

Josefine Nebl[1], Jan Philipp Schuchardt[1], Paulina Wasserfurth[1], Sven Haufe[2], Julian Eigendorf[2], Uwe Tegtbur[2], Alexander Ströhle[1] and Andreas Hahn[1]*

[1] Faculty of Natural Sciences, Institute of Food Science and Human Nutrition, Leibniz University Hannover, 30159 Hannover, Germany; nebl@nutrition.uni-hannover.de (JN); schuchardt@nutrition.uni-hannover.de (JPS); wasserfurth@nutrition.uni-hannover.de (PW); hahn@nutrition.uni-hannover.de (AH)

[2] Institute of Sports Medicine, Hannover Medical School, 30625 Hannover, Germany; Haufe.Sven@mh-hannover.de (SH); Eigendorf.Julian@mh-hannover.de (JE); Tegtbur.Uwe@mh-hannover.de (UT)

* Correspondence: hahn@nutrition.uni-hannover.de; Tel.: +49-511-762-5093

nutrients

MDPI

Table S 1. Biomarkers of iron status and hematological parameters according to gender.

Parameter		**Omnivores** n=27	**p value Omnivores *vs.* Lacto-Ovo**	**Lacto-ovo** n=26	**p value Lacto-Ovo *vs.* Vegan**	**Vegan** n=28	**p value Omnivores *vs.* Vegan**	**p value**
Vitamin B_{12}, pmol/l	f	302±116	-	345±134	-	324±301	-	0.273[b]
	m	353±127	-	281±188	-	311±145	-	0.317[b]
Deficient (< 150 pmol/l), n (%)	f	1 (4)		0 (0)		2 (7)		
	m	0 (0)		2 (8)		1 (4)		
Holo-TC, pmol/l	f	80.8±32.3	n.s.	79.8±28.0	0.042[c]	67.9±39.6	n.s.	0.047[a]
	m	79.8±28.0	-	68.1±34.4	-	67.7±41.1	-	0.662[a]
Deficient (< 35 pmol/l), n (%)	f	1 (4)		1 (4)		4 (14)		
	m	0 (0)		1 (4)		2 (7)		
MMA, nmol/l	f	270±181	-	253±171	-	448±703	-	0.687[b]
	m	253±171	-	331±227	-	209±73	-	0.062[b]
Deficient (> 271 nmol/l), n (%)	f	3 (11)		2 (8)		5 (18)		
	m	2 (7)		4 (15)		2 (7)		
tHcy, µmol/l	f	11.5±3.37	-	13.2±1.85	-	12.0±3.52	-	0.514[b]
	m	13.2±1.85	-	16.5±8.37	-	14.4±5.20	-	0.462[b]
> 10 µmol/l, n (%)	f	1 (4)		3 (12)		3 (11)		
	m	2 (7)		6 (23)		3 (11)		
4cB12	f	0.90	-	1.15	-	0.66	-	0.148[a]
	m	0.92	-	0.53	-	0.77	-	0.359[a]

f = female, m = male, Holo-TC = holotranscobalamin, MMA = methylmalonic acid, 4cB12 = 4 markers combined vitamin B-12 indicator [25], n.s. = not significant, tHcy = total homocysteine. Values are given as means ± SD or n (%) of the study population in the different cut-off values. [a] One-way ANOVA, [b] Kruskal Wallis test, [c] Post Hoc test.

Table S 2. Biomarkers of vitamin D status according to gender.

Parameter		Omnivores n=27	Lacto-ovo n=26	Vegan n=28	p value
25(OH)D, nmol/l	f	98.2±30.9	85.0±39.5	87.4±40.0	
	m	79.5±31.9	65.5±21.4	88.0±41.0	0.516[a]
Optimal	f	12 (44)	8 (31)	9 (32)	0.308[a]
(≥75 nmol), n (%)	m	6 (22)	3 (12)	7 (25)	
Sufficiency	f	4 (15)	4 (15)	5 (18)	
(50-74.9 nmol/l), n (%)	m	2 (7)	5 (19)	0	
Insufficiency	f	0	3 (12)	2 (7)	
(25-49.9 nmol/l), n (%)	m	3 (11)	2 (8)	2 (7)	
Deficiency	f	0	0	1 (4)	
(<25 nmol/l), n (%)	m	0	0	1 (4)	

f = female, m = male, 25(OH)D = 25-hydroxyvitamin D. Values are given as means ± SD or n (%) of the study population in the different cut-off values. [a] One-way ANOVA.

.

Table S 3. Biomarkers of iron status and hematological parameters according to supplement intake.

Parameter		**Omnivores** n=27	**Lacto-ovo** n=26	**Vegan** n=28	**p value**
Iron serum, µmol/l	SU	19.4±4.74	12.6±3.58	13.1±4.99	0.277[a]
	non-SU	17.5±8.43	18.7±7.91	17.5±6.38	0.839[a]
Deficiency	SU	0 (0)	1 (4)	1 (4)	
(<10 µmol/l), n (%)	non-SU	7 (26)	4 (15)	1 (4)	
Ferritin, µg/l	SU	59.0±24.0	32.7±19.5	40.8±22.4	0.408[b]
	non-SU	62.6±57.5	40.4±34.7	45.7±33.7	0.706[b]
Depleted iron stores	SU	0 (0)	0 (0)	1 (4)	
(< 15 µg/l), n (%)	non-SU	7 (26)	6 (23)	3 (11)	
Transferrin, µmol/l	SU	36.4±1.78	44.8±4.04	36.4±1.78	0.061[b]
	non-SU	42.1±12.2	39.7±6.88	40.4±7.53	0.957[b]
Increased iron requirement	SU	0 (0)	1 (4)	0 (0)	
(≥ 47.7 µmol/l), n (%)	non-SU	6 (22)	3 (12)	3 (11)	
Transferrin saturation	SU	26.5±4.95	14.0±4.36	17.8±6.65	0.129[a]
	non-SU	23.2±14.3	24.4±11.1	23.1±10.4	0.913[a]
Insufficient iron supply	SU	0 (0)	1 (4)	2 (7)	
(< 16%), n (%)	non-SU	10 (37)	5 (19)	7 (25)	
Hb, g/dl	SU	13.9±1.48	14.9±0.76	13.5±0.59	0.166[a]
	non-SU	13.9±1.42	14.0±1.04	14.1±1.47	0.825[a]
Anemia	SU	3 (11)	0 (0)	4 (14)	
(< 12.0/13.0 g/dl), n (%)	non-SU	0 (0)	1 (4)	0	
Hct, l/l	SU	0.41±0.04	0.45±0.02	0.40±0.02	0.087[a]
	non-SU	0.41±0.04	0.41±0.03	0.43±0.04	0.263[a]
< 0.36 (f)/0.39 (m), n (%)		0 (0)	0 (0)	0 (0)	
MCV, fl	SU	87.9±4.03	91.0±2.80	88.0±4.00	0.543[a]
	non-SU	87.4±3.79	88.8±4.59	89.0±3.91	0.365[a]
Iron deficiency anemia (< 80 fl), n (%)		0 (0)	0 (0)	0 (0)	

SU = supplement-users, non-SU = non-supplement users, MCV = Mean Corpuscular Volume. Values are given as means ± SD or n (%) of the population in the different cut-off values. [a] One-way ANOVA, [b] Kruskal Wallis test.
